# Efficacy Assessment of Nucleic Acid Decontamination Reagents Used in Molecular Diagnostic Laboratories

**DOI:** 10.1371/journal.pone.0159274

**Published:** 2016-07-13

**Authors:** Melina Fischer, Nathalie Renevey, Barbara Thür, Donata Hoffmann, Martin Beer, Bernd Hoffmann

**Affiliations:** 1 Institute of Diagnostic Virology, Friedrich-Loeffler-Institut, Südufer 10, 17493, Greifswald-Insel Riems, Germany; 2 Institute of Virology and Immunology, Sensemattstr, 293, 3147, Mittelhäusern, Switzerland; University of Quebec at Trois-Rivieres, CANADA

## Abstract

The occurrence of nucleic acid cross contamination in the laboratory resulting in false positive results of diagnostic samples is seriously problematic. Despite precautions to minimize or even avoid nucleic acid cross contaminations, it may appear anyway. Until now, no standardized strategy is available to evaluate the efficacy of commercially offered decontamination reagents. Therefore, a protocol for the reliable determination of nucleic acid decontamination efficacy using highly standardized solution and surface tests was established and validated. All tested sodium hypochlorite-based reagents proved to be highly efficient in nucleic acid decontamination even after short reaction times. For DNA Away, a sodium hydroxide-based decontamination product, dose- and time-dependent effectiveness was ascertained. For two other commercial decontamination reagents, the phosphoric acid-based DNA Remover and the non-enzymatic reagent DNA-ExitusPlus^™^ IF, no reduction of amplifiable DNA/RNA was observed. In conclusion, a simple test procedure for evaluation of the elimination efficacy of decontamination reagents against amplifiable nucleic acid is presented.

## Introduction

In recent years, polymerase chain reaction (PCR) has been used frequently for various applications in molecular biology, inter alia for the diagnosis of infectious agents [1]. Especially when it comes to diagnostics the minimization of nucleic acid cross contaminations is of utmost importance. Despite precautions to avoid contaminations, such as spatial separation of PCR preparation from product handling, ultraviolet (UV) irradiation or uracil-N-glycosylase treatment [2–4], nucleic acid cross contaminations may occur in molecular diagnostic laboratories anyway. The crucial question in these situations is: Which reagents are most efficient in decontaminating nucleic acid? Recently, Champlot et al. [5] have established a decontamination strategy for PCR reagents, but a standardized protocol for assessment of the efficacy of decontamination reagents in the molecular diagnostic laboratory is missing. Standardized national and international guidelines are available for determination of the efficacy of disinfectants [6, 7], while for the evaluation of decontamination reagents such specifications are missing. In the present study the efficacy of four commercial nucleic acid decontamination reagents used in molecular laboratories was compared with a hypochlorite-containing domestic cleaning agent and a freshly prepared 1% sodium hypochlorite solution as reference substance. Decontamination is defined as degradation, denaturation or inactivation of amplification of nucleic acids. Targets for the comparative analyses were short PCR amplicons (target DNA) and *in vitro* transcripts (target RNA) as the most relevant cross contaminants in a routine PCR lab. In this context the reduction of amplifiable target DNA or RNA by the different decontamination reagents was investigated independently of the principle of operation. Based on the disinfection guideline of the German Veterinary Society [8] and in compliance with European norms for suspension tests [9] and surface tests [10] we propose a test strategy for the evaluation of nucleic acid decontamination reagents. The recommended test procedure might be helpful for molecular diagnostic laboratories to verify the efficacy of decontamination reagents.

## Materials and Methods

### Generation of target nucleic acids (`cross contamination`)

For generation of both target nucleic acids (DNA-amplicon and *in-vitro* RNA) used in this study the pGEM-EGFP2rev plasmid, established by Hoffmann et al. [11] (containing a 712 bp fragment of the EGFP gene), was utilized.

The DNA-amplicon (named target DNA) was produced by amplification of a 712 bp fragment of the EGFP gene from the pGEM-EGFP2rev plasmid using the QuantiTect Multiplex PCR NoRox Kit (Qiagen, Hilden, Germany). For one reaction 5.5 μL RNase-free water, 12.5 μL 2x QuantiTect Multiplex PCR Master Mix, 1.0 μL of primers EGFP-15-F and EGFP-10-R (10 μM) [11] each, as well as 5.0 μL pGEM-EGFP2rev plasmid (2x 10^9^ copies per μL) were mixed. The following thermal program was applied: 1 cycle of 95°C for 15 min, followed by 45 cycles of 95°C for 60 s, 60°C for 30 s, and 72°C for 30 s. PCR products were analyzed on a 1.5% agarose gel and purified using the QIAquick Gel Extraction Kit (Qiagen) according to the manufacturer's instructions. The exact number of DNA molecules was calculated as described [12] and adjusted to a concentration of 2x 10^7^ copies per μL. Dilution of the target DNA was performed in phosphate buffered saline (PBS).

For generation of the target RNA, the pGEM-EGFP2rev plasmid was linearized using the restriction enzyme *NcoI* and agarose gel was purified as described by Hoffmann et al. [11]. The resulting plasmid DNA was *in vitro* transcribed using the RiboMAX Large Scale RNA Production Systems (Promega, Mannheim, Germany). A DNase I digestion was performed subsequently using the SP6/T7 Transcription Kit (Roche Diagnostics, Mannheim, Germany) according to the manufacturer’s instructions. During purification of the *in vitro* transcribed RNA using the RNeasy Mini Kit (Qiagen) a second on-column DNase I digestion according to the manufacturer’s recommendations was implemented. The exact number of RNA molecules was calculated as described [12] and adjusted to a concentration of 2x 10^7^ copies per μL. The target RNA was diluted in RNase free water.

### Decontamination reagents

As reference substance a 1% sodium hypochlorite solution was prepared from a 10–15% stock solution (Sigma-Aldrich Chemie GmbH, Steinheim, Germany). Relevant data regarding the applied commercial decontamination reagents are summarized in Table 1. All reagents were used before the date of expiry. Dilutions of the decontamination reagents were prepared freshly in 1x PBS (testing of target DNA) or RNase free water (testing of target RNA) before application. Appropriate storage conditions and storage life were considered for the reagents tested.

**Table 1:** Set of decontamination reagents applied in this study.

Reagent	Manufacturer	Lot number	Residence time [min]^#^	Reactive component	Tested dilutions
Hypochlorite (10–15%)	Sigma-Aldrich Chemie GmbH, Steinheim, Germany	STBD5198V	-	sodium hypochlorite	1% solution, 1:4, 1:16
DNA Away	Carl Roth GmbH & Co. KG, Karlsruhe, Germany	12441062	1	sodium hydroxide	undil., 1:4, 1:16
DNA Remover	Minerva Biolabs GmbH, Berlin, Germany	152D1082	1	phosphoric acid	undil., 1:4, 1:16
DNA-ExitusPlus^™^ IF	AppliChem GmbH, Darmstadt, Germany	3P002304	10	non enzymatic	undil., 1:4, 1:16
LTK-008	BioDelta GmbH, Löhne, Germany	-	5–10	sodium hypochlorite	undil., 1:4, 1:16
Sagrotan Schimmel-frei*	Reckitt Benckiser, Mannheim, Germany	-	10–15	sodium hypochlorite	undil., 1:4, 1:16

^#^: recommended by the supplier

*: identical with Complete Clean Mould & Mildew Remover from DETTOL (Reckitt Benckiser; Berkshire, UK)

-: no information available; undil.: undiluted reagent

### Solution test

Suspension tests (based on Chemical disinfectants and antiseptics—Quantitative suspension test for the evaluation of bactericidal activity of chemical disinfectants and antiseptics used in veterinary area—Test method and requirements (DIN EN 1656) [9]) were performed as preliminary approach to gain basic information and to facilitate comparison of the efficacy of the different decontamination reagents. Therefore, 10 μL of the respective reagent or a dilution of the reagent (indicated in Table 1) were filled into a well of MagNA Pure LC Processing Cartridges (Roche Diagnostics, Mannheim, Germany) in triplicates. Furthermore, wells with 10 μL of 1x PBS or RNase free water were implemented as no-reagent-controls and delivered the reference Cq values for the target DNA and RNA of each experiment. To start the decontamination procedure, 10 μL of the target nucleic acid were added to the well; the solution was mixed thoroughly and spun down. Reactions of target DNA or RNA with the decontamination reagents were stopped after 2 or 10 min. For this purpose, 180 μL 1x PBS and 200 μL lysis buffer AL (MagAttract Virus Mini M48 Kit from Qiagen) were added to each well together with 10 μL internal control nucleic acid, i.e. T7-DNA (20–50 pg; GeneOn, Ludwigshafen, Germany) or MS2-RNA (80 pg; Roche Diagnostics) to ensure proper RNA/DNA-extraction and the performance of real-time (reverse transcriptase (RT-))-PCR. The solution was mixed thoroughly by shaking, spun down and then incubated for 15 min at room temperature. After incubation, the content of each well was transferred to a Thermo 96-well deep well plate (Thermo Scientific, Braunschweig, Germany) and nucleic acid was extracted using the MagAttract Virus Mini M48 Kit (Qiagen) according to the manufacturer’s instructions on the King Fisher 96 Flex (Thermo Scientific) platform. All tests were performed as triplicates twice on two independent days. Moreover, an undiluted aliquot of each tested decontamination reagent was kept un-protected from light for two weeks at room temperature and was then tested again with the target DNA to investigate a light-induced loss of efficacy. All incubations were performed at room temperature (21±1°C).

### Surface test

Surface tests resembling laboratory conditions (based on the procedure described in Chemical disinfectants and antiseptics—Quantitative surface test for the evaluation of bactericidal activity of chemical disinfectants and antiseptics used in the veterinary area on non-porous surfaces without mechanical action—Test method and requirements (DIN EN 14349) [10]) were performed by drying 10 μL target nucleic acid in MagNA Pure LC Processing Cartridges (Roche Diagnostics) in triplicates overnight at room temperature in a laminar hood. Afterwards 50 μL of the respective decontamination reagent, reagent dilution or no-reagent-control (see solution test) were added and incubated for 2 or 10 min. Decontamination reactions were stopped by adding 150 μL 1x PBS and 200 μL lysis buffer AL (MagAttract Virus Mini M48 Kit from Qiagen). Also here 10 μL internal control nucleic acid (T7-DNA or MS2-RNA) were added to each well and the nucleic acid was extracted as described above.

### Real-time PCR

For amplification and detection of the respective target (`cross contamination`) and the internal control nucleic acid, the Takyon No ROX Probe Mastermix Kit (Eurogentec, Köln, Germany) was used. Briefly, for amplification of the target DNA and the internal DNA control 1.0 μL RNase-free water, 5.0 μL Takyon MasterMix, 1.0 μL EGFP-Mix1 (Table 2), 1.0 μL internal control DNA mix (Table 2) and 2.0 μL template, positive control or RNase free water for the no template control (NTC) were used per reaction. The following thermal program was applied: 95°C for 3 min, followed by 40 cycles of 95°C for 10 s, 57°C for 20 s and 68°C for 30 s. For reverse transcriptase (RT-) PCR of the target RNA and the internal RNA control the Takyon No ROX Probe Mastermix Kit (Eurogentec) was used in combination with the RT core kit (Eurogentec). For one reaction 0.85 μL RNase-free water, 5.0 μL Takyon MasterMix, 0.05 μL Euroscript RT mix, 0.1 μL RT additive, 1.0 μL EGFP-Mix1 (Table 2), 1.0 μL internal control RNA mix (Table 2) and 2.0 μL template, positive control or RNase free water for the no template control (NTC) were mixed. The following thermal program was applied: 1 cycle of 48°C for 30 min and 95°C for 3 min, followed by 40 cycles of 95°C for 10 s, 57°C for 20 s and 68°C for 30 s.

**Table 2:** Oligonucleotide sequences and composition of primer-probe mixes used in this study.

Detection of	Assay	Primer/Probe	Sequence (5'-3')	Concentration [μM]
	EGFP-Mix1	EGFP1-F	GAC CAC TAC CAG CAG AAC AC	2.5
target RNA and DNA	(amplify a 133 bp fragment)	EGFP2-R	GAA CTC CAG CAG GAC CAT G	2.5
		EGFP-Probe 1	FAM- AGC ACC CAG TCC GCC CTG AGC A -BHQ1	1.25
	T7-DNA-Mix6	T7-14510-F	GCG GTC TTA TTG TGT TCC AC	7.5
internal control DNA	(amplify a 86 bp fragment)	T7-14595-R	GAA CTC TCG GTT CAA TTG CAA C	7.5
		T7-14535-HEX	HEX- TCA CAA GTA TGA CGT TCC TGC ATT GAC -BHQ1	1.88
	MS2-RNA-Mix10	MS2-3167-F	GCT ACT CGC GGA TAC CCG	7.5
internal control RNA	(amplify a 123 bp fragment)	MS2-3289-R	ACT TCA CCT CCA GTA TGG AAC	7.5
		MS2-TM3-HEX	HEX- ACC TCG GGT TTC CGT CTT GCT CGT -BHQ1	1.88

FAM: 6-carboxyfluorescein; HEX: 5′-hexachlorofluorescein; BHQ1: black hole quencher 1.

All reactions were performed as triplicates in Bio-Rad 96-well PCR plates using a CFX96 quantitative PCR system (Bio-Rad Laboratories, Hercules, USA). For each real-time PCR, a quantification cycle number (C_q_) was determined according to the PCR cycle number at which the fluorescence of the reaction exceeds a value that is statistically higher than the background, which is determined by the respective software associated with the system.

## Results and Discussion

To assess the efficacy of five commercial nucleic acid decontamination reagents used in the laboratory as well as a domestic cleaning agent (Table 1) a double-check strategy, composed of a solution test and a surface test, was performed (Tables 3 and 4). As a first step, the solution test was established to enable a highly standardized comparison [6] of the different products. In this approach equal volumes of the decontamination reagent and the target nucleic acid (i.e. DNA-amplicon or *in-vitro* RNA) were mixed and incubated for 2 or 10 min, respectively. The reaction was stopped by adding PBS and the lysis buffer intended from the nucleic acid extraction kit. The capability of these combined reagents to stop the reaction was confirmed in preliminary tests (data not shown). Nucleic acid was extracted and amplified by real-time (RT-) PCR. Negative effects of the different decontamination reagents regarding the inhibition-free nucleic acid extraction and real-time (RT-) PCR amplification were excluded by integration of internal process controls (T7-DNA and MS2-RNA).

**Table 3:** Evaluation of the nucleic acid decontamination efficacy by the solution test protocol.

		DNA-amplicon				*in-vitro* RNA			
Reaction time		2 min		10 min		2 min		10 min	
Reagent	Dilution	mean C_q_	± SD	mean C_q_	± SD	mean C_q_	± SD	mean C_q_	± SD
no reagent	undil.	17.8	0.40	17.5	0.25	19.1	0.11	19.6	0.49
1% Hypochl.	all	no C_q_	-	no C_q_	-	no C_q_	-	no C_q_	-
	undil.	35.4	0.98	no C_q_	-	no C_q_	-	no C_q_	-
DNA Away	1:4	32.2	1.30	36.9	0.68	no C_q_	-	no C_q_	-
	1:16	20.2	0.89	23.6	0.23	34.7	1.30	no C_q_	-
	undil.	17.2	0.07	17.8	0.30	19.7	0.40	19.5	0.14
Remover	1:4	17.3	0.12	18.3	0.36	19.7	0.41	19.9	0.34
	1:16	18.0	0.29	17.7	0.37	19.7	0.29	19.7	0.34
	undil.	17.1	0.20	16.9	0.13	18.5	0.26	18.4	0.26
DNA Exitus	1:4	17.4	0.37	17.7	0.42	18.8	0.13	18.8	0.12
	1:16	17.6	0.50	18.6	0.77	19.1	0.11	19.1	0.18
LTK-008	all	no C_q_	-	no C_q_	-	no C_q_	-	no C_q_	-
Sagrotan	all	no C_q_	-	no C_q_	-	no C_q_	-	no C_q_	-

no reagent: no-reagent-control; 1% Hypochl.: 1% hypochlorite solution (reference substance); Remover: DNA Remover; DNA Exitus: DNA-ExitusPlus^™^ IF; Sagrotan: Sagrotan Schimmel-frei; undil.: undiluted; all: no C_q_ for all dilution values; mean C_q_: mean C_q_ value from 6 replicates; SD: standard deviation; no C_q_: no C_q_-value detected; -: no value available.

**Table 4:** Evaluation of the nucleic acid decontamination efficacy by the surface test protocol.

		DNA-amplicon				*in-vitro* RNA			
Reaction time		2 min		10 min		2 min		10 min	
Reagent	Dilution	mean C_q_	± SD	mean C_q_	± SD	mean C_q_	± SD	mean C_q_	± SD
no reagent	undil.	24.0	0.29	23.2	0.58	21.2	0.34	21.3	1.28
1% Hypochl.	all	no C_q_	-	no C_q_	-	no C_q_	-	no C_q_	-
	undil.	36.2	0.64	36.1	0.44	no C_q_	-	no C_q_	-
DNA Away	1:4	32.4	0.29	33.7	0.38	no C_q_	-	no C_q_	-
	1:16	30.8	0.66	31.3	0.32	no C_q_	-	no C_q_	-
	undil.	23.6	0.67	23.6	0.65	22.5	1.49	21.9	1.17
Remover	1:4	24.8	0.57	24.2	0.58	21.4	0.65	21.7	0.36
	1:16	24.2	0.46	23.8	0.50	22.1	0.86	21.0	0.66
	undil.	24.3	0.39	24.2	0.43	21.3	0.51	21.4	0.91
DNA Exitus	1:4	23.1	0.39	22.9	0.58	20.5	0.95	20.6	0.40
	1:16	23.2	1.11	23.9	0.81	21.5	1.44	21.4	0.67
LTK-008	all	no C_q_	-	no C_q_	-	no C_q_	-	no C_q_	-
Sagrotan	all	no C_q_	-	no C_q_	-	no C_q_	-	no C_q_	-

no reagent: no-reagent-control; 1% Hypochl.: 1% hypochlorite solution (reference substance); Remover: DNA Remover; DNA Exitus: DNA-ExitusPlus^™^ IF; Sagrotan: Sagrotan Schimmel-frei; undil.: undiluted; all: no C_q_ for all dilution values; mean C_q_: mean C_q_ value from 6 replicates; SD: standard deviation; no C_q_: no C_q_-value detected; -: no value available.

After just a short treatment (2 min) with the reference substance (1% hypochlorite solution, Sigma-Aldrich Chemie) or with other hypochlorite-based reagents, i.e. LTK-008 (BioDelta) and Sagrotan Schimmel-frei (Reckitt Benckiser), neither target DNA nor target RNA were amplifiable by (RT-) PCR (no C_q_) for all tested decontamination reagent dilutions (Table 3). A hypochlorite solution was used as reference substance because it is easy to prepare, cheap and widely used in molecular laboratories [13, 14]. Furthermore, Prince and Andrus [14] demonstrated its high decontamination efficacy. In our approach, all tested hypochlorite-based products, including the domestic cleaning agent, seem to be highly efficient. In contrast, the phosphoric acid-based DNA Remover (Minerva Biolabs, Berlin, Germany) and the non-enzymatic reagent DNA-ExitusPlus^™^ IF (AppliChem, Darmstadt, Germany) did not show any reduction of amplifiable nucleic acid. Even after a 10 min treatment with the undiluted products, detected C_q_ values were similar to those measured from the no-reagent-control.

The sodium hydroxide-based DNA Away reagent (Carl Roth, Karlsruhe, Germany) displayed a dose-dependent efficacy. Incubation for 2 min with DNA Away led to partial elimination of the target DNA, indicated by a prominent increase of the C_q_ values in comparison to the no-reagent-control (Table 3). The decontamination efficacy increased from the 1:16 dilution up to the undiluted reagent (1:16<1:4<undil.). After a 10 min treatment using undiluted DNA Away, no target DNA was detectable anymore. Furthermore, DNA Away exhibited an improved performance of target RNA elimination. No RNA was detectable after a 2 min incubation using the undiluted product and the 1:4 dilution. After incubation for 10 min, no RNA target was detectable at all.

Finally, undiluted aliquots of all decontamination reagents as well as the reference substance were tested again after a 14-day storage period at room temperature, un-protected from light. Despite these non-appropriate storage conditions, differences regarding the decontamination performance for DNA were not observed (Table 5). Especially, the light sensitive hypochlorite-based reagents remained highly efficient.

**Table 5:** Effects of the storage of decontamination reagents on the DNA decontamination efficacy of the undiluted reagents.

Reaction time		2 min		10 min	
Reagent	Light	mean C_q_	± SD	mean C_q_	± SD
no reagent	-	16.8	0.19	16.9	0.22
1% Hypochl.	+	no C_q_	-	no C_q_	-
	-	no C_q_	-	no C_q_	-
DNA Away	+	35.5	0.19	no C_q_	-
	-	35.6	0.09	no C_q_	-
Remover	+	16.6	0.24	16.6	0.24
	-	16.9	0.32	17.0	0.31
DNA Exitus	+	17.0	0.10	17.0	0.13
	-	17.3	0.16	17.0	0.17
LTK-008	+	no C_q_	-	no C_q_	-
	-	no C_q_	-	no C_q_	-
Sagrotan	+	no C_q_	-	no C_q_	-
	-	no C_q_	-	no C_q_	-

no reagent: no-reagent-control; 1% Hypochl.: 1% hypochlorite solution (reference substance); Remover: DNA Remover; DNA Exitus: DNA-ExitusPlus^™^ IF; Sagrotan: Sagrotan Schimmel-frei; mean C_q_: mean C_q_ value from 3 replicates; SD: standard deviation; no C_q_: no C_q_-value detected; -: no value available; Storage of the decontamination reagents 14 days at room temperature +: un-protected from light; -: protected from light.

As a second step, the more practice-orientated surface test was analyzed. In this approach actually 50 μL reagent were applied to the dried up target nucleic acid to ensure complete coverage of the dried `contamination`. Again incubation times of 2 min or 10 min, respectively, were tested and the nucleic acid decontamination efficacy was analyzed by real-time PCR. Analogous to the solution test, the reference substance, LTK-008 and Sagrotan Schimmel-frei eliminated amplifiable nucleic acid, DNA as well as RNA, in every tested dilution after a 2 min treatment (Table 4). DNA Remover and DNA-ExitusPlus^™^ IF again were ineffective yielding similar C_q_ values as the no-reagent-control. With regard to the surface test, DNA Away was not as effective in eliminating the DNA target as in the solution test (Tables 3 and 4). Even after a 10 min treatment using DNA Away, only partial dose-dependent elimination of the target DNA was observed. However, the dried up target RNA was eliminated effectively by every tested dilution already after 2 min of incubation. Non-template controls were always negative.

In general, real-time (RT-) PCR analysis of the internal control nucleic acid (T7 DNA or MS2 RNA) revealed no extraction failure or PCR inhibition in the analyzed reactions (concerning solution test and surface test), as evident by the consistent C_q_ values measured from all samples (S1 and S2 Tables). Due to nucleic acid degradation during the drying process as well as re-dissolving effects, obtained C_q_ values are generally increased in the surface test compared with the solution test despite of using the same amount of starting material.

All incubation steps of DNA/ RNA and controls in the presence of decontamination reagent were performed in Magna Pure plates to ensure optimal contact between the target and the reagent accounted for by the V-shaped design of the wells. Due to this well design standardized and reproducible results can be achieved even when using low volumes. The reproducibility observed for three extractions per dilution on two independent days (n = 6 samples) resulted in low standard deviation (SD) values (Tables 3 and 4). Moreover, the plate design makes it easy to perform the 2 min and the 10 min incubation separately. This separation improves handling during the experiment, and based on the highly reproducible real-time (RT-) PCR data as well as the design of the experiments cross contamination is most widely excluded.

In summary, all tested hypochlorite-based reagents convinced by their high efficacy of nucleic acid decontamination in the solution test as well as in the surface test, even after short incubation periods. The sodium hydroxide-containing decontamination reagent showed a dose- and time-dependent reduction of amplifiable nucleic acid. These results were similar to results from Champlot and co-workers [5]. The authors used bleach and DNA Away for elimination of DNA contaminations from surfaces. In contrast, the decontamination reagents based on phosphoric acid (DNA Remover) or non-enzymatic compounds (DNA-ExitusPlus^™^ IF) did not reduce the load of amplifiable DNA or RNA according to our test procedure. To the best of our knowledge, the decontaminating potential of DNA Remover and DNA-ExitusPlus^™^ IF determined in comprehensive tests has not been published yet. Studies dealing with the efficacy of DNA Remover were not found at all. Esser and co-workers (2006) [15] presented results for the functionality of DNA Exitus^™^ and DNA-ExitusPlus^™^ IF for degradation of plasmid DNA in a sponsored paper. Furthermore, Arena (2010) [16] compared DNA-ExitusPlus^™^ IF to a 10% sodium hypochlorite solution in order to reduce human DNA on different work surfaces. This insufficiently controlled study indicated an in-complete decontamination effect for both reagents.

Most of the tested products are identified as harmful to human health due to irritating and/ or corrosive effects. Thus the recommended safety measures should be followed when using these products. Only DNA-ExitusPlus^™^ IF is described to be not harmful to human health and non-toxic. While the safety at work would benefit from non-harmful substances in general, effective elimination of amplifiable RNA or DNA molecules must be achieved to be suitable for application as decontamination reagent.

The prevention and destroying of contaminating nucleic acids is very important for diagnostic and research laboratories operating with conventional and novel molecular methods. Incorrect assumptions, based on contaminating RNA or DNA, might result in erroneous individual diagnostic reports, but can be also the origin for the misinterpretation of established databases. Recently, Cantalupo and co-workers (2015) [17] identified nucleic acid contaminations originating from human papillomavirus 18 intrinsic to HeLa cells in non-cervical samples of The Cancer Genome Atlas (TCGA) database and concluded that nucleic acid contamination occurs frequently during experimentation at the bench. Therefore, the use of functional decontaminating reagents helps to reduce false sequence information extensively.

Surprisingly, clear differences in the elimination efficacy of the different decontamination reagents against amplifiable nucleic acids were observed based on the presented simple test systems. It should be mentioned that very short PCR products, representing standard amplicons of a routine diagnostic PCR lab, were amplified in the presented study and stronger decontamination effects to high-molecular nucleic acids by the different non-hypochlorite reagents cannot be excluded.

However, the aim of the presented study was to establish a standardized protocol for testing of decontamination agents based on the disinfection guideline of the German Veterinary Society [8] as well as the European norms for suspension [9] and surface tests [10]. This protocol is easily applicable for the manufacturer and/or user to confirm the functionality of decontamination reagents. In addition, we recommend that manufacturers include information of ingredient substances and decontamination capacity. In analogy to disinfection agents decontamination reagents would benefit from the indicating an effectiveness value against standardized indicator DNA.

## Supporting Information

S1 Table Evaluation of the internal control nucleic acid by the solution test protocol. (DOCX)

S2 Table Evaluation of the internal control nucleic acid by the surface test protocol. (DOCX)
