# Supplementary material for: Efficacy Assessment of Nucleic Acid Decontamination Reagents Used in Molecular Diagnostic Laboratories
Source: PLoS One. 2016 Jul 13;11(7):e0159274. doi: 10.1371/journal.pone.0159274 (PMC4943653; doi:10.1371/journal.pone.0159274)
Supplement: S1 Table — (DOCX) [file pone.0159274.s001.docx]

**S1 Table.** Evaluation of the internal control nucleic acid by the solution test protocol.

|  | | DNA-amplicon | | | | *in-vitro* RNA | | | |
| --- | --- | --- | --- | --- | --- | --- | --- | --- | --- |
| Reaction time | | 2 min | | 10 min | | 2 min | | 10 min | |
| Reagent | Dilution | mean C_q_ | ± SD | mean C_q_ | ± SD | mean C_q_ | ± SD | mean C_q_ | ± SD |
| no reagent | undil. | 24.2 | 0.47 | 24.1 | 0.33 | 25.2 | 0.19 | 26.1 | 0.57 |
|  | undil. | 24.3 | 0.45 | 24.4 | 0.10 | 25.5 | 0.44 | 26.0 | 1.20 |
| 1% Hypochl. | 1:4 | 24.1 | 0.35 | 24.3 | 0.28 | 25.6 | 0.43 | 25.5 | 0.29 |
|  | 1:16 | 24.6 | 0.60 | 25.1 | 0.74 | 25.4 | 0.67 | 25.4 | 0.33 |
|  | undil. | 24.2 | 0.20 | 24.5 | 0.50 | 25.3 | 0.62 | 25.5 | 0.33 |
| DNA Away | 1:4 | 24.5 | 0.30 | 25.1 | 0.56 | 25.4 | 0.49 | 25.2 | 0.73 |
|  | 1:16 | 23.8 | 0.46 | 23.7 | 0.32 | 25.3 | 0.50 | 25.4 | 0.55 |
|  | undil. | 23.8 | 0.17 | 24.6 | 0.64 | 24.9 | 0.62 | 24.7 | 0.40 |
| Remover | 1:4 | 24.1 | 0.52 | 25.6 | 0.57 | 24.7 | 0.59 | 24.9 | 0.51 |
|  | 1:16 | 24.7 | 0.52 | 23.9 | 0.47 | 24.8 | 0.52 | 25.0 | 0.72 |
|  | undil. | 23.7 | 0.28 | 23.8 | 0.27 | 24.3 | 0.22 | 24.3 | 0.32 |
| DNA Exitus | 1:4 | 23.9 | 0.43 | 24.0 | 0.46 | 25.1 | 0.12 | 24.8 | 0.24 |
|  | 1:16 | 24.0 | 0.49 | 24.9 | 0.76 | 25.2 | 0.15 | 25.0 | 0.21 |
|  | undil. | 24.2 | 0.53 | 24.9 | 0.83 | 25.6 | 0.54 | 25.3 | 0.25 |
| LTK-008 | 1:4 | 24.7 | 0.51 | 25.3 | 0.18 | 26.2 | 0.08 | 25.6 | 0.29 |
|  | 1:16 | 24.6 | 0.51 | 24.5 | 0.32 | 26.1 | 0.30 | 25.7 | 0.35 |
|  | undil. | 25.1 | 0.26 | 25.4 | 0.66 | 25.9 | 0.89 | 25.9 | 0.31 |
| Sagrotan | 1:4 | 25.4 | 0.78 | 25.6 | 1.12 | 25.8 | 0.22 | 26.2 | 0.35 |
|  | 1:16 | 25.3 | 0.65 | 25.0 | 0.66 | 25.8 | 0.22 | 26.2 | 0.28 |

no reagent: no-reagent-control; 1% Hypochl.: 1% hypochlorite solution (reference substance); Remover: DNA Remover; DNA Exitus: DNA ExitusPlus^TM^ IF; Sagrotan: Sagrotan Schimmel-frei; undil.: undiluted; mean C_q_: mean C_q_ value from 6 replicates; SD: standard deviation.
