# Supplementary material for: Efficacy Assessment of Nucleic Acid Decontamination Reagents Used in Molecular Diagnostic Laboratories
Source: PLoS One. 2016 Jul 13;11(7):e0159274. doi: 10.1371/journal.pone.0159274 (PMC4943653; doi:10.1371/journal.pone.0159274)
Supplement: S2 Table — (DOCX) [file pone.0159274.s002.docx]

**S2 Table.** Evaluation of the internal control nucleic acid by the surface test protocol.

|  | | DNA-amplicon | | | | *in-vitro* RNA | | | |
| --- | --- | --- | --- | --- | --- | --- | --- | --- | --- |
| Reaction time | | 2 min | | 10 min | | 2 min | | 10 min | |
| Reagent | Dilution | mean C_q_ | ± SD | mean C_q_ | ± SD | mean C_q_ | ± SD | mean C_q_ | ± SD |
| no reagent | undil. | 25.3 | 0.44 | 25.1 | 0.39 | 24.9 | 0.33 | 25.7 | 1.10 |
|  | undil. | 24.6 | 0.25 | 24.7 | 0.15 | 25.6 | 0.53 | 25.9 | 0.63 |
| 1% Hypochl. | 1:4 | 24.6 | 0.38 | 25.1 | 0.80 | 25.5 | 0.38 | 25.2 | 0.31 |
|  | 1:16 | 24.9 | 0.56 | 25.8 | 0.45 | 25.7 | 1.01 | 25.1 | 0.26 |
|  | undil. | 24.7 | 0.49 | 25.2 | 0.69 | 24.7 | 0.40 | 24.3 | 0.31 |
| DNA Away | 1:4 | 24.6 | 0.56 | 25.6 | 0.24 | 25.1 | 0.26 | 24.9 | 0.30 |
|  | 1:16 | 24.4 | 0.40 | 24.8 | 0.96 | 25.0 | 0.27 | 24.9 | 0.33 |
|  | undil. | 24.7 | 0.39 | 24.6 | 0.52 | 25.0 | 0.20 | 24.5 | 0.27 |
| Remover | 1:4 | 25.2 | 0.71 | 25.3 | 0.29 | 24.6 | 0.24 | 24.5 | 0.44 |
|  | 1:16 | 25.1 | 0.69 | 24.9 | 0.54 | 24.7 | 0.37 | 24.4 | 0.40 |
|  | undil. | 24.7 | 0.29 | 24.7 | 0.29 | 24.2 | 0.08 | 24.1 | 0.29 |
| DNA Exitus | 1:4 | 24.0 | 0.32 | 24.5 | 0.48 | 24.8 | 0.72 | 24.3 | 0.13 |
|  | 1:16 | 24.4 | 0.39 | 25.4 | 0.36 | 24.7 | 0.43 | 24.6 | 0.18 |
|  | undil. | 24.5 | 0.21 | 25.4 | 0.54 | 25.2 | 0.25 | 25.2 | 0.25 |
| LTK-008 | 1:4 | 25.0 | 0.47 | 25.8 | 0.54 | 25.3 | 0.33 | 25.4 | 0.09 |
|  | 1:16 | 24.8 | 0.45 | 25.6 | 0.80 | 25.4 | 0.10 | 25.3 | 0.48 |
|  | undil. | 24.8 | 0.53 | 25.9 | 0.65 | 25.4 | 0.33 | 25.5 | 0.33 |
| Sagrotan | 1:4 | 25.8 | 0.73 | 26.1 | 0.55 | 25.0 | 0.39 | 25.6 | 0.36 |
|  | 1:16 | 25.4 | 0.31 | 25.4 | 0.47 | 25.6 | 0.24 | 25.5 | 0.41 |

no reagent: no-reagent-control; 1% Hypochl.: 1% hypochlorite solution (reference substance); Remover: DNA Remover; DNA Exitus: DNA ExitusPlus^TM^ IF; Sagrotan: Sagrotan Schimmel-frei; undil.: undiluted; mean C_q_: mean C_q_ value from 6 replicates; SD: standard deviation.
